# Association Between Nutrients and Cardiovascular Diseases

**DOI:** 10.2174/011573403X263414231101095310

**Published:** 2024-01-05

**Authors:** Amir Shakarami

**Affiliations:** 1 Department of Cardiology, Faculty of Medicine, Lorestan University of Medical Sciences, Khorramabad, Iran

**Keywords:** Cardiovascular diseases (CVD), carbohydrates, micro-nutrients, vitamins, inflammatory, oxidative stress

## Abstract

Cardiovascular diseases (CVD) constitute a leading cause of global mortality. Inflammation and oxidative stress are key molecular underpinnings of CVD pathogenesis. This comprehensive review explores the multifaceted role of nutrients in cardiovascular health beyond their impact on cardiac events. The manuscript examines the influence of macronutrients such as fats and carbohydrates, as well as micronutrients including vitamins and folate, on CVD. Additionally, the interplay between dietary supplements and CVD risk reduction is investigated. The purpose of this manuscript is to provide a comprehensive overview of the diverse mechanisms through which nutrients contribute to cardiovascular well-being, addressing both cardioprotective effects and their broader implications. Through an analysis of pertinent studies, we illuminate the complex relationship between nutrition, lifestyle, and cardiovascular health, underscoring the significance of a holistic approach to CVD prevention and management.

## 1 INTRODUCTION

Cardiovascular diseases (CVD) have been reported as a leading cause of death in the United States for over 8 decades [1]. Approximately, 17.3 million deaths per annum are due to CVD [2]. Some of the common mechanisms involved in cardiovascular diseases include oxidative stress and inflammation [3, 4]. The most evident risk factors of CVD include hyperlipidemia, hypertension, advanced age, smoking, hypertriglyceridemia, and diabetes [5].

Groundbreaking work [6] initiated a great number of research investigating the role of diet and nutrients in CVD. Randomized controlled trials and cohort studies have consistently demonstrated that unhealthy dietary habits can contribute to the risk of cardiovascular diseases [7]. Conversely, the consumption of dietary supplements has shown potential in mitigating the likelihood of adverse cardiovascular events [8]. Despite molecular evidence and the beneficial effects of these supplements, discrepancies in clinical findings have been reported. Consumption of vegetables, nuts, and the Mediterranean diet can have cardioprotective effects [9]. Furthermore, moderate evidence has been found regarding vitamins, monosaturated fatty acids, folate, alcohol, and fruits [10]. The effects of diet and nutrients have been extensively associated with CVD risk such as thrombosis, inflammation, hyperlipidemia, hypertension, insulin sensitivity, and vascular dysfunction [11, 12]. According to a study by Nurses’ Health Studies, lifestyle, diet, and physical activity are important factors contributing to cardiovascular health [13]. Approximately 40% of premature CVD deaths can be controlled by the modification of lifestyle [14].

This review provides details regarding various macro and micro-nutrients, their cardiovascular effects, and associated molecular evidence.

## 2 MICRONUTRIENTS

### 2.1 Vitamin B

Tryptophan metabolism is known to be associated with an active form of vitamin B6, pyridoxal phosphate, (PLP). Deficiency of PLP is extensively reported to be associated with an increased incidence of CVD [15]. Whereas, tryptophan load and kynurenine to tryptophan ratio reduction as a result of increased tryptophan breakdown in heart disease patients have been widely associated with CVD [16].

Increased tryptophan catabolite and xanthurenic acid excretion is marked by low vitamin B6 status. The formation of kynurenic acid, xanthurenic acid, 3′-hydroxy anthranilic acid, and anthranilic acid is associated with plasma concentration of vitamin B6 [17]. Fig. (**1**) illustrates the involvement of Vitamin B6 in the tryptophan metabolism. This pathway involves the conversion of tryptophan, an essential amino acid, into various metabolites including kynurenine and its derivatives. Vitamin B6 acts as a coenzyme in the enzymatic reactions that facilitate the conversion of tryptophan to kynurenine.

Several pro-inflammatory cytokines like interferon-γ (INF-γ) mediate the tryptophan metabolism, indicating the role of the immune system in tryptophan catabolism [18]. In atherosclerosis, inflammation is manifested by increased proinflammatory cytokines like INF-γ and increased tryptophan catabolism.

A recent prospective cohort study conducted in China reported that dietary intake of vitamin B6 reduces the risk of mortality and causes cardiovascular mortality, like stroke and coronary artery disease [19]. Vitamin B has been specifically indicated to reduce the risk of stroke [20]. In a meta-analysis, [21] researchers concluded that a combined intake of vitamin B6, B12, and folic acid supplements might not reduce the risk of myocardial infarction; however, it can maintain the homocysteine levels and the risk of stroke [22]. Vitamin D is known for its role in bone health and calcium regulation. However, recent research suggests that it may also play a role in modulating homocysteine levels. According to a study published in the Journal of Clinical Endocrinology & Metabolism, vitamin D may contribute to the maintenance of optimal homocysteine concentrations through its involvement in the one-carbon metabolic pathway [23].

**Fig. (2):**
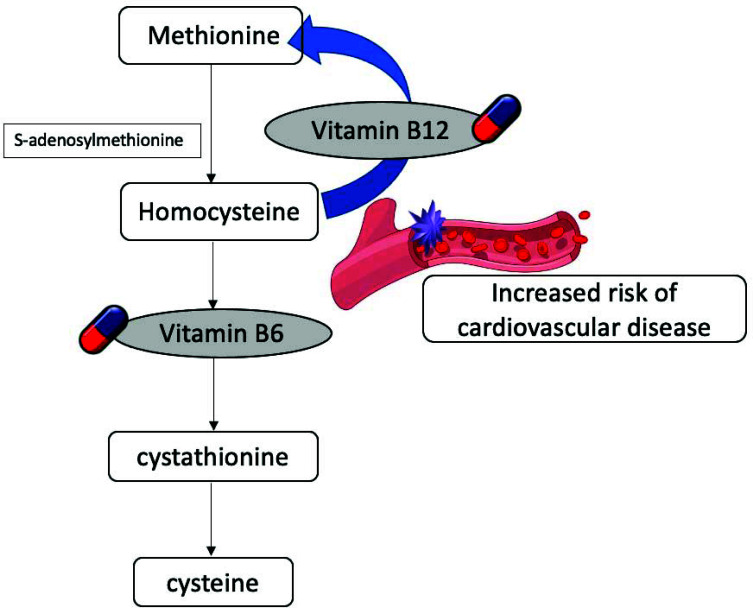
The role of vitamin B6 and vitamin B12 in hyperhomocysteinemia.

Vitamin B12 acts as a co-factor along with methyltetrahydrofolate for the remethylation of homocysteine to methionine. Homocysteine is an amino acid that cannot be obtained from diet; however, methionine is consumed from indigested dietary products [24]. In the case of increased methionine, transsulphuration of homocysteine takes place [25]. Vitamin B6 (pyridoxine) and vitamin B12 (cobalamin) play crucial roles in the metabolism of homocysteine, exerting a significant influence on the hyperhomocysteinemia pathway (Fig. **2**).

Homocysteine imposes damage to vascular epithelium and elevates platelet adhesiveness. It increases oxidative stress and leads to atherosclerosis. Among CVD patients, hyperhomocysteinemia is associated with increased oxidative stress and reduced high density lipoprotein-cholesterol [26]. Similarly, increased homocysteine levels are positively correlated with hypertension [27] and congenital heart disease. Hydrogen sulfide increases neovascular activity and reduces oxidative stress. Conversion of homocysteine to hydrogen sulfide is, thus, known to have cardioprotective effects [28]. A recent study reported that homocysteine and c-reactive protein not only mark chronic inflammation among CVD patients but is also associated with increased incidence of systemic lupus erythematous [29]. Additionally, several clinical studies have indicated that vitamin B12 can reduce homocysteine levels and the corresponding risk of CVD [30]. Low levels of vitamin B12 are associated with increased obesity and dyslipidemia, corresponding to increased levels of triglycerides, cholesterol, tumor necrosis factor-α, interleukin 1b, and interleukin 6 [31]. From recent findings by hyperhomocysteinemia and low levels of vitamin B6 are positively associated with all-cause mortality whereas reduced homocysteine IL-6 and c-reactive protein and increased vitamin B6 levels are presented with longer telomeres [32]. Telomerase shortening because of inflammation and oxidative stress are also, purposed mechanisms of the role of vitamin B deficiency in the pathogenesis of CVD. Vitamin B12 deficiency among vegetarians might also impose the risk of CVD [33]. Furthermore, the Mediterranean diet leads to decreased homocysteine levels and hence might offer a cardioprotective advantage [34].

### 2.2 Vitamin D

Activation of vitamin D is characterized by 25-hydroxylation in the liver, converted into 25-hydroxyvitamin D (25OHD) and 1,25-dihydroxyvitamin D (1,25(OH)2D), vitamin D hormone, in the kidney. Briefly, the metabolism of vitamin D is regulated by parathyroid hormone, calcium, phosphate, and fibroblast growth factor-23 (FGF-23) [35]. Vitamin D has been long known for the pathogenesis of CVD. Seasonal variations in the incidence of cardiovascular events and related mortality have been primary evidence indicating the protective role of vitamin D. Vitamin D receptors (VDR) are widely expressed in extra-osseous tissue [36-38]. In the cardiovascular system, expression of VDR has been reported in ventricular myocytes and fibroblasts, endothelium of veins and capillaries, and vascular smooth muscles [39].

Renin-angiotensin-aldosterone system (RAAS) plays important role in hypertension. Studies have shown that VDR activation downregulates a number of elements of RAAS, such as renin, renin receptors, angiotensin II type 1 receptors, and angiotensinogen [40]. Reduced vitamin D level also increases pulse pressure and decreases collagen, elastin, and endothelial nitric oxide synthetase [41, 42]. Overall, vitamin D modulates vascular tone and vitamin D deficiency increases oxidative stress and cyclooxygenase-1 [43-45]. Systemic inflammation due to infection in vitamin D-deficient patients can also increase the risk of hypertension [46, 47]. A recent study concluded that vitamin D deficiency is not only associated with an increased risk of pulmonary arterial hypertension but is also correlated with poor prognosis and mortality. However, a recent systematic review by the Centers for Disease Control and Prevention, including 11 cohort studies and 27 randomized control trials, concluded that vitamin D supplementations are not associated with a reduction in blood pressure [48]. Similar findings have been concluded by another systematic study [49]. Vitamin D also regulates gene expression of homocysteine metabolism enzymes; therefore, vitamin D deficiency can impair homocysteine metabolism [50]. A study reported that vitamin D supplements can lower homocysteine levels, body weight, and body mass index (BMI) among CVD patients [51].

The generation of macrophage-derived foam cells plays a key role in the pathogenesis of atherosclerosis [52]. Inflammation due to vitamin D deficiency has also been extensively studied [53]. Proinflammatory and inflammatory cytokines like c-reactive protein, IL-6, TNF-α, and nuclear factor-κB (NF‐κB) modulate inflammatory response in atherosclerosis and coronary artery disease. Vitamin D is recognized to have an anti-inflammatory response, therefore having cardioprotective effects [54]. Fig. (**3**) illustrates the immune-mediated cardioprotective effects of vitamin D, highlighting its role in modulating the immune response within the cardiovascular system.

Vascular smooth muscle cell-derived tissue factor is critical for arterial thrombosis and inflammation, mediated by vitamin D deficiency, along with upregulation of NF‐κB and TNF-α increases the production of tissue factor [55]. One of the serious complications of atherosclerosis plaques is the blockage of a coronary artery that leads to myocardial infarction and stroke. Deficiency of vitamin D is a significant indicator of ST-elevation myocardial infarction in acute coronary syndrome patients [56]. In a recent 10-year cohort study, following the dietary intake of vitamin D (fish and egg) incidence of CVD was significantly reduced in men as a result of decreased inflammatory parameters such as c-reactive protein, IL-6, and fibrinogen [57].

Vitamin D has anti-fibrotic and anti-hypertrophic properties that can prevent left ventricular hypertrophy [58]. Animal studies have shown that vitamin D treatment improves left ventricular wall thickness, end-diastolic and systolic volume left ventricular ejection fraction, fractional shortening and gene expression of atrial natriuretic peptide and brain natriuretic peptide [59]. Suppression of PARP1 (Poly (ADP-ribose) polymerase 1)/SIRT1 (Sirtuin 1)/mTOR (mammalian target of rapamycin) in response to vitamin D treatment is the suggested mechanism [60].

### 2.3 Folate/Folic Acid

A meta-analysis of randomized control trials by [61] concluded that folic acid supplements reduces the incidence of cardiovascular event among chronic kidney disease patients in a dose-dependent manner. Similar to vitamin B, folic acid therapy reduces serum homocysteine levels. A cohort study on 10-year National Health and Nutrition Examination Survey (NHANES) data reported that reduced folate levels were associated with increased risk of all-cause mortality and cardiovascular mortality [62]. This relationship was more prominent in the older population [63].

**Fig. (4):**
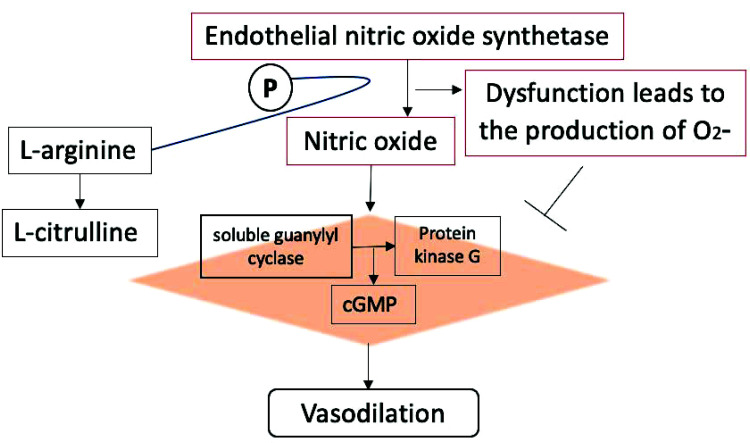
Endothelial nitric oxide (eNOS) activation mediates the production of L-citrulline and nitric oxide with the help of co-factors. Nitric oxide diffuses the endothelial into vascular smooth muscle where it leads to the activation of protein kinase G by soluble guanylyl cyclase, causing vasodilation. Oxidative stress causes eNOS uncoupling and endothelial dysfunction which favors the production of O2- instead of nitric oxide.

Endothelial dysfunction, characterized by vasoconstriction, increased platelet aggregation and adhesion, inflammation and elevation in smooth muscle activity, has been extensively reported to be associated with nitric oxide (NO) deficiency. Nitric oxide is one of the important vasodilators where its reduction is not only associated with vasoconstriction but also leads to inflammatory response as a result of increased production of intracellular adhesion molecule-1, vascular adhesion molecule-1, E-selectin and macrophage chemoattractant peptide-1. Furthermore, it has anti-thrombotic effects and smooth muscle cell proliferation. Increased oxidative stress decreases the production of nitric oxide and causes endothelial dysfunction (Fig. **4**) [40, 78]. As mentioned above, similar to vitamin B6 and B12, folate is important for homocysteine metabolism and its deficiency is associated with elevation of homocysteine levels. Homocysteine reduces the production of nitric oxide and leads to endothelial nitric oxide decoupling by protein kinase C activation and is therefore known to contribute to atherosclerosis, ischemic heart disease, stroke and thromboembolic disease [64, 65]. A recent literature review showed that folate supplements can correct hyperhomocysteinemia and can have protective effects against coronary heart disease [66]. However, both high and low levels of red blood cells (RBCs) folate increase the risk of abdominal aortic calcification [67]. Furthermore, its prescription during pregnancy should be carefully monitored as it can increase the risk of gestational diabetes [68, 69].

### 2.4 Vitamin A

Vitamin A is well-known for immunity [70] and has protective effects against a number of pathologies such as depression [71], inflammatory bowel disease [72], infectious and autoimmune diseases [73] and multiple sclerosis [74, 75], to name a few. Retinoids inhibit Th17 cell-mediated pro-inflammatory response by the action of retinoid-related orphan receptor-c (RORc). RORc is an important transcription factor that regulates the differentiation and proliferation of Th17 along with other transcription factors to produce IL-17. Retinoic acid upregulates the production of TGF-β (tissue growth factor-β) and Foxp3 (forkhead boxp3) and downregulates that of Th-17 and the production of IL-17 [76, 77]. The emerging role of IL-17 in the pathogenesis of atherosclerosis has been extensively reported now [78]. A study has shown that vitamin A supplementation significantly reduces RORc gene expression and IL-17 production, which is likely to have protective effects against atherosclerosis [79]. A number of clinical and preclinical studies have indicated that vitamin A can reduce the risk of atherosclerosis-associated cardiovascular disease. Nonetheless, some of the findings are contrary to this [80].

Additionally, retinoic acid suppresses Th1 response and polarization of M1 macrophages whereas increased Th-1 response favors atheroma by increasing the production of IL-12 and INF-γ. On the other hand, vitamin A induces Th2-mediated anti-inflammatory response by increasing the production of IL-6 and suppressing that of INF-γ. It also promotes M2 macrophage production [81].

The released form of vitamin A, retinol, binds to retinol-binding protein 4 (RBP4), which binds to transthyretin in blood. Target tissue converts retinol into retinoic acid that transcribes a number of genes, particularly those targeting lipid metabolism and lipoprotein [82]. It binds to apolipoprotein B100 (apoB) containing LDL, which increases the production of foam cells, progressing the formation of atherosclerosis plagues [83]. Vitamin A levels have a dose-dependent effect on the cardiovascular system and reduced levels of vitamin A are associated with an increased risk of all-cause mortality [84]. It also affects serum cholesterol and triglyceride levels [85]. However, systemic reviews and meta-analyses have concluded that the pieces of evidence regarding the prevention of cardiovascular disease with vitamin A supplements are limited and might not have clinical efficacy [86, 87].

Some of the common mechanisms involved in cardiovascular diseases include oxidative stress and inflammation [3, 4].

Some studies have indicated that vitamin B and vitamin E supplements do not necessarily lead to risk-reducing effects on cardiovascular diseases (CVD) [86]. However, these outcomes could be influenced by confounding variables or inadequate study design. Moreover, it's important to consider that the simultaneous use of certain medications, such as statins, folic acid, and acetylsalicylic acid, may impact the interpretation of these findings.

### 2.5 Vitamin E

Vitamin E classifies eight naturally occurring compounds under its umbrella, where RRR-α-tocopherol is the most abundant form of lipid-soluble vitamin E found in the cellular and sub-cellular membrane that is known for its anti-oxidant properties. Nonetheless, dietary γ-tocopherol is a more effective antioxidant against nitrogen species than α-tocopherol [88]. Additionally, vitamin E is seen to have an anti-inflammatory effect by inhibiting the production of NF-κB in a dose-dependent manner. Nucleotide‐binding domain and leucine‐rich repeat pyrin domain inflammasome complex are triggered in response to NF-κB activation and oxidative stress. Increased stress response and production of NF-κB due to vitamin E deficiency activates inflammasome [89]. A recent *ex-vivo* study showed that treatment of vitamin E and melatonin in homocysteine-induced apoptosis endothelial cells reduces the levels of reactive oxidation species and lipid peroxide along with suppression of apoptotic proteins like caspases 3, 9, cytochrome C and Bax and enhances the production of Bcl2. Furthermore, it increases cell migration. Overall, the treatment was effective against homocysteine-induced cell damage [90].

Epidemiological diversity has been extensively reported when evaluating the cardioprotective role of vitamin E. Some studies have reported that vitamin E reduces the risk of myocardial infarction and angina pectoris; some report a greater incidence of coronary artery disease in relation to vitamin E, whereas others have shown no association [91]. Patients with sub-clinical atherosclerosis show significant improvement in peripheral artery disease [92].

### 2.6 Vitamin C

Vitamin C (ascorbic acid) is a water-soluble vitamin that is known for its antioxidant and anti-inflammatory properties. Vitamin C inhibits the formation of atherosclerotic plaque by preventing oxidation of LDL. Similar to folate and other vitamins, it enhances the production of nitric oxide. It also inhibits the production of reactive oxygen species and monocyte aggregation on endothelial walls [93]. Vitamin C supplements in hypertensive and/or diabetic obese adults are responsible for a significant reduction in CRP, IL-6, blood glucose and triglyceride levels [94]. Furthermore, a randomized control trial concluded that vitamin C supplements improve endothelial dysfunction in atherosclerotic, diabetic and heart failure patients [95]. A recent study by [96] on the levels of vitamin C and antioxidant profile in saliva of coronary correlated with elevated serum C-reactive protein among these patients. A systemic review and meta-analysis also concluded that higher vitamin C intake and greater levels of blood vitamin C and E and β-carotene reduce the risk of CVD-related death [97].

### 2.7 Macronutrients

#### 2.7.1 Fats

Fats are the type of triglycerides where their toxicity (levels more than normal) is considered as harmful lipids. Excessive exposure to lipids leads to impairment of lipid metabolism in endoplasmic reticulum (ER) that mediates the alteration in the phospholipid membrane of ER. Increased lipotoxicity (increased fatty acids and cholesterol load) induces oxidative stress and activates inflammatory response. When the capacity to store fats in white adipose tissue is exhausted, fats are deposited into other organs like heart, muscle, liver and pancreas, where these lipid particles activate immune response in the tissue [98].

Dietary saturated fat has been a long-known risk factor for cardiovascular pathologies [99]. An increase in low-density lipoprotein is important to manage atherosclerosis. Demographic studies have shown that the Mediterranean and Asian population has a low prevalence of CVD due to reduced saturated fat in their diet. The traditional Mediterranean diet involves a high intake of olive oil, nuts, fruits and vegetables, moderate consumption of fish and poultry, and low consumption of processed and red meat and sweets. A study showed that Mediterranean diet with nuts or extra virgin olive oil significantly reduces the risk of CVD than individuals taking a low-fat diet (composed of low-fat dairy products, bread, potato, pasta, rice, fruits, vegetables and seafood) [100, 101]. The impact of dietary fats on heart physiology comes from early animal studies, which reported increased serum cholesterol levels to cause arterial lesions [102], which has been later verified by a number of clinical studies and randomized control trials. However, within the last few years, it has been reported that not all the patients suffering from CVD are presented with increased total cholesterol and variability in lipid profile is likely to occur [103–105]. Primarily, in order to manage CVD, an imbalance in cholesterol levels is targeted, which is recommended by the replacement of saturated fat with polyunsaturated fat. Furthermore, emphasis on the elimination of trans-fatty acids from the diet is also advised since they are rich in LDL and have a lesser amount of high-density lipoprotein [106]. A Cochrane review [107] concluded that reduction of saturated fat intake is positively related to the decreased risk of CVD, where the replacement of saturated with polyunsaturated fat is beneficial Omega-3 is a polyunsaturated fatty acid and is known to have a therapeutic effect against inflammation, oxidative stress and hyperlipidemia among diabetic and CVD patients [108]. However, a recent Cochrane review suggested that long-chain Omega 3 supplements do not reduce the risk of all-cause mortality, stroke and arrhythmia and may slightly reduce the risk of coronary heart disease events and mortality, respectively [109].

Dyslipidemia is a common presentation among CVD patients and is considered an important biomarker to predict prognosis [110]. Despite statin therapy being well-known to reduce LDL-cholesterol, remaining elevated triglyceride levels also impose the risk and exacerbation of atherosclerosis. Triglyceride-rich lipoprotein (TGRL) can easily cross the arterial wall and is likely to be retained by connective tissue matrix due to interface with the positively charged residues (arginine and lysine) on apoB (apolipoprotein B). LDL particles undergo oxidative modification so they can bind to scavenger receptors on macrophages, causing the formation of foam cells and thereby leading to the production of atherosclerotic plaque [111]. TGRL leads to endothelial dysfunction by inducing oxidative stress and inflammation mediated by increased production of TNF-α, IL-6, Il-8 IL-1β and proatherogenic adhesion molecules [112, 113]. A number of clinical studies have indicated that elevated triglycerides are associated with an increased risk of CVD [114]. Nordestgaard *et al.* [115] reported that increased non-fasting triglycerides were correlated with remnant lipoprotein cholesterol and were positively associated with increased incidence of myocardial infarction, ischemic heart disease, and mortality in men and women [116].

## 3 CARBOHYDRATES

For short-term weight loss, a low carbohydrate diet with high protein and fat intake has been practiced. However, long-term effect of carbohydrate intake on cardiovascular health remains disputed. A recent cohort study and meta-analysis showed that 50–55% energy consumption from carbohydrates is associated with the lowest risk of mortality, whereas low carbohydrate intake <40% and high intake >70% is associated with a high risk of all-cause mortality [117]. Though the replacement of saturated fatty acids with carbohydrates has been counseled to prevent and reduce the progression of CVD, a number of researches have shown no improvement or exacerbation of the disease [111, 118, 119,]. This is because adverse outcomes associated with atherogenic dyslipidemia are characterized by increased triglyceride, small and dense LDL-cholesterol and reduced HDL-cholesterol [120]. The Prospective Urban Rural Epidemiology (PURE) study enrolling 18 countries showed that a high intake of carbohydrates leads to an increased risk of all-cause mortality, whereas the risk of CVD and cardiovascular mortality is not affected [121].

Excessive intake of starch can convert sugar into fats (*de novo* lipogenesis). The process multiplies to several folds when carbohydrates are taken more than the body’s energy requirement. The chief products of *de novo* lipogenesis include palmitate, saturated fatty acids and monounsaturated fatty acids [122]. Hence, a high intake of carbohydrates can impair glucose oxidation and is associated with increased serum LDL and triglycerides. Recent data from the China Health and Nutrition Survey reported that intake of carbohydrates is negatively associated with total cholesterol and triglycerides in men and total cholesterol and LDL in women. Nonetheless, it was positively correlated with waist circumference, body mass index, and blood pressure in women. Therefore, moderate intake of carbohydrates is only recommended [123].

## CONCLUSION

Substantial shreds of evidence have related nutrients and diet to cardiovascular diseases; however, it appears that patient-specific conditions and the nature of the disease are likely to affect these outcomes. Several reports and meta-analyses have invalidated the overall beneficial effects of supplements in these patients [86]. The severity of the disease, genetics, environmental factors and co-morbidities might have an impact on these outcomes. Though a balanced diet is recommended with low saturated fatty acids in healthy individuals, diet modifications are required in CVD patients, which should be provided by clinicians and regularly followed up. Overall intake of nutrients in the recommended amount might reduce the incidence of cardiovascular disease, nonetheless, therapeutic benefits are yet to be fully deciphered.

## Figures and Tables

**Fig. (1):**
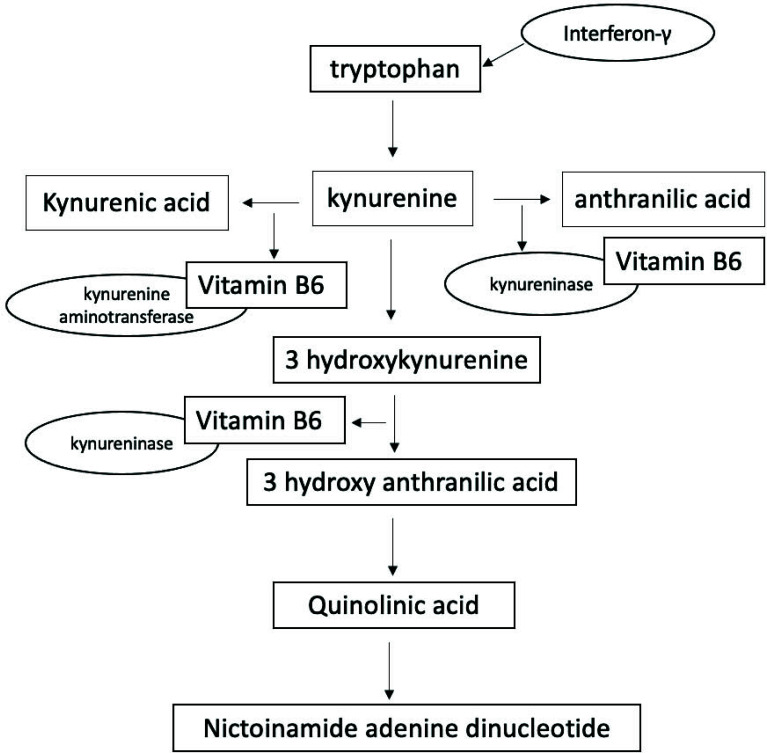
Formation of kynurenic acid, xanthurenic acid, 3′-hydroxy anthranilic acid, and anthranilic acid associated with plasma concentration of vitamin B6 (36).

**Fig. (3):**
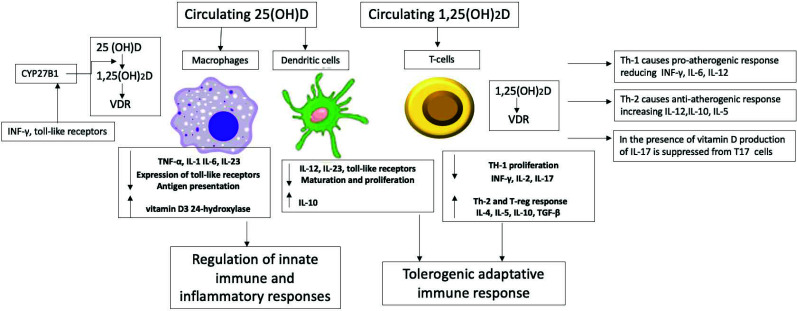
The figure illustrates the immune-mediated cardioprotective effects of vitamin D.

## LIST OF ABBREVIATIONS

1,25(OH)2D: 1,25-Dihydroxyvitamin D
25OHD: 25-Hydroxyvitamin D
BMI: Body Mass Index
CRP: C-Reactive Protein
CVD: Cardiovascular Diseases
ER: Endoplasmic Reticulum
FGF-23: Fibroblast Growth Factor-23
HDL: High-Density Lipoprotein
IL: Interleukin
INF-γ: Interferon Gamma
LDL: Low-Density Lipoprotein
LDL-cholesterol: Low-Density Lipoprotein Cholesterol
mTOR: Mammalian Target of Rapamycin
NF-κB: Nuclear Factor-Kappa B
PARP1: Poly (ADP-Ribose) Polymerase 1
PURE: Prospective Urban Rural Epidemiology Study
RAAS: Renin-Angiotensin-Aldosterone System
RBP4: Retinol-Binding Protein 4
RRR-α-tocopherol: All-rac-α-tocopherol (form of vitamin E)
SIRT1: Sirtuin 1
TGRL: Triglyceride Rich Lipoprotein
Th1: T-helper 1 cells
Th17: T-helper 17 cells
Th2: T-helper 2 cells
VDR: Vitamin D Receptor
